# Effused and Confused: A Case of Severe Adenovirus Pneumonia in a Teen

**DOI:** 10.7759/cureus.75165

**Published:** 2024-12-05

**Authors:** Ilias E Dimeas, Stratos Skrimizeas, George Dimeas, Irini Gerogianni, Zoe Daniil

**Affiliations:** 1 Department of Respiratory Medicine, Faculty of Medicine, University of Thessaly, University General Hospital of Larissa, Larissa, GRC; 2 Department of Internal Medicine, General Hospital of Karditsa, Karditsa, GRC

**Keywords:** adenoviral infection, antibiotic escalation, community-acquired pneumonia (cap), human adenovirus, pcr testing, pleural effusion, severe pneumonia, unresponsive pneumonia, viral pathogens

## Abstract

Adenovirus is a common pathogen responsible for respiratory infections, including pneumonia, predominantly in pediatric populations but also in immunocompromised adults. This case report details an 18-year-old immunocompetent male presenting with severe lobar pneumonia and pleural effusion, initially suggesting a bacterial origin. Despite antibiotic treatment, the patient's symptoms persisted, prompting further investigation. The diagnostic approach included polymerase chain reaction testing on upper-airway specimens, pleural effusion, and bronchoalveolar lavage, which identified adenovirus as the causative agent. This diagnosis confirmed that adenoviral pneumonia could present with radiologic findings similar to bacterial infections, such as lobar consolidation and pleural effusion, which are typically considered suggestive of bacterial etiology. The successful identification of adenovirus from pleural effusion, previously reported in bronchoalveolar lavage or upper-airway specimens, is a novel aspect of this case, underscoring the importance of comprehensive diagnostic testing, especially in atypical or severe cases that fail to respond to empiric therapy. Antimicrobial therapy was continued to prevent secondary bacterial infections, but the primary treatment was supportive care. The patient showed significant clinical improvement and was discharged in stable condition after seven days, with no residual symptoms at follow-up. This case highlights the need for careful consideration of viral pathogens, particularly adenovirus, in the differential diagnosis of severe pneumonia, even in the presence of pleural effusion, to avoid unnecessary antibiotic escalation and guide appropriate management. Early viral identification and tailored treatment strategies are essential, particularly in cases that do not respond to conventional therapies. Furthermore, this case emphasizes the value of advanced diagnostic techniques in identifying less common pathogens and reducing the risks of unnecessary antibiotic use.

## Introduction

Adenovirus, a double-stranded DNA virus from the Adenoviridae family, is a known pathogen responsible for a broad spectrum of illnesses, including respiratory infections such as pneumonia [1]. While adenoviral pneumonia is primarily observed in children, particularly those under five, it can also affect adults, especially those with compromised immune systems [2]. In pediatric populations, adenovirus is a significant contributor to respiratory disease, accounting for around 4.7% of severe pneumonia cases in children under five years old [3].

The clinical presentation of adenoviral pneumonia varies widely, from mild bronchopneumonia to life-threatening conditions such as acute respiratory distress syndrome (ARDS) [4]. Typical symptoms include fever, cough, and difficulty breathing; however, severe cases may require hospitalization and carry high mortality rates, especially among young children. Although adenovirus infections are typically self-limiting in immunocompetent individuals, they can lead to severe complications such as ARDS, myocarditis, hepatitis, encephalitis, and multi-organ dysfunction syndrome (MODS). In untreated cases, mortality rates can exceed 50%, highlighting the importance of supportive care to manage the disease while the immune system clears the infection [5, 6].

Adenovirus spreads through aerosolized droplets, conjunctival inoculation, and fecal-oral transmission. Contaminated surfaces can also serve as vectors. The incubation period is two to 14 days, with the infection site determined by the route of entry, typically respiratory for droplets [7].

Though typically self-limiting, adenoviral pneumonia may resemble bacterial pneumonia in severe cases, complicating diagnosis due to overlapping radiographic findings like lobar consolidation and pleural effusion [8]. This radiological similarity poses a significant diagnostic challenge, highlighting the importance of comprehensive viral testing to differentiate adenoviral infections from bacterial etiologies [9] as well as non-common pathogens [10]. In cases where clinical findings initially suggest bacterial pneumonia but the patient remains unresponsive to antibiotics, a thorough viral workup becomes essential to avoid unnecessary escalation of antimicrobial therapy and to explore potential immunocompromised status [11].

Our case report describes an 18-year-old patient who presented with severe lobar pneumonia and pleural effusion caused by adenovirus, initially suggesting a bacterial origin. What makes this case unique is the successful isolation of adenovirus in the pleural effusion using polymerase chain reaction (PCR), which was pivotal in confirming the viral etiology. This diagnostic achievement underscores the importance of considering viral pathogens, such as adenovirus, in the differential diagnosis of severe pneumonia, even when pleural effusion is present. Advanced diagnostic techniques, including upper-airway and bronchoalveolar lavage (BAL) film arrays and pleural fluid PCR, were crucial in guiding appropriate management, preventing unnecessary antibiotic use, and prompting investigation for possible underlying immunocompromise. The case is particularly relevant to current clinical practice, as it highlights the growing role of PCR in diagnostics and the importance of antibiotic stewardship in the management of severe pneumonia.

## Case presentation

An 18-year-old male patient, a non-smoker, was referred to the Emergency Department with a six-day history of persistent fever. His illness had begun a week earlier with a fever reaching up to 39.4°C, dry cough, appetite loss, and general malaise. Outpatient treatment with azithromycin and amoxicillin/clavulanate for three days failed to improve his symptoms. He had no notable past medical history.

**Figure 1:**
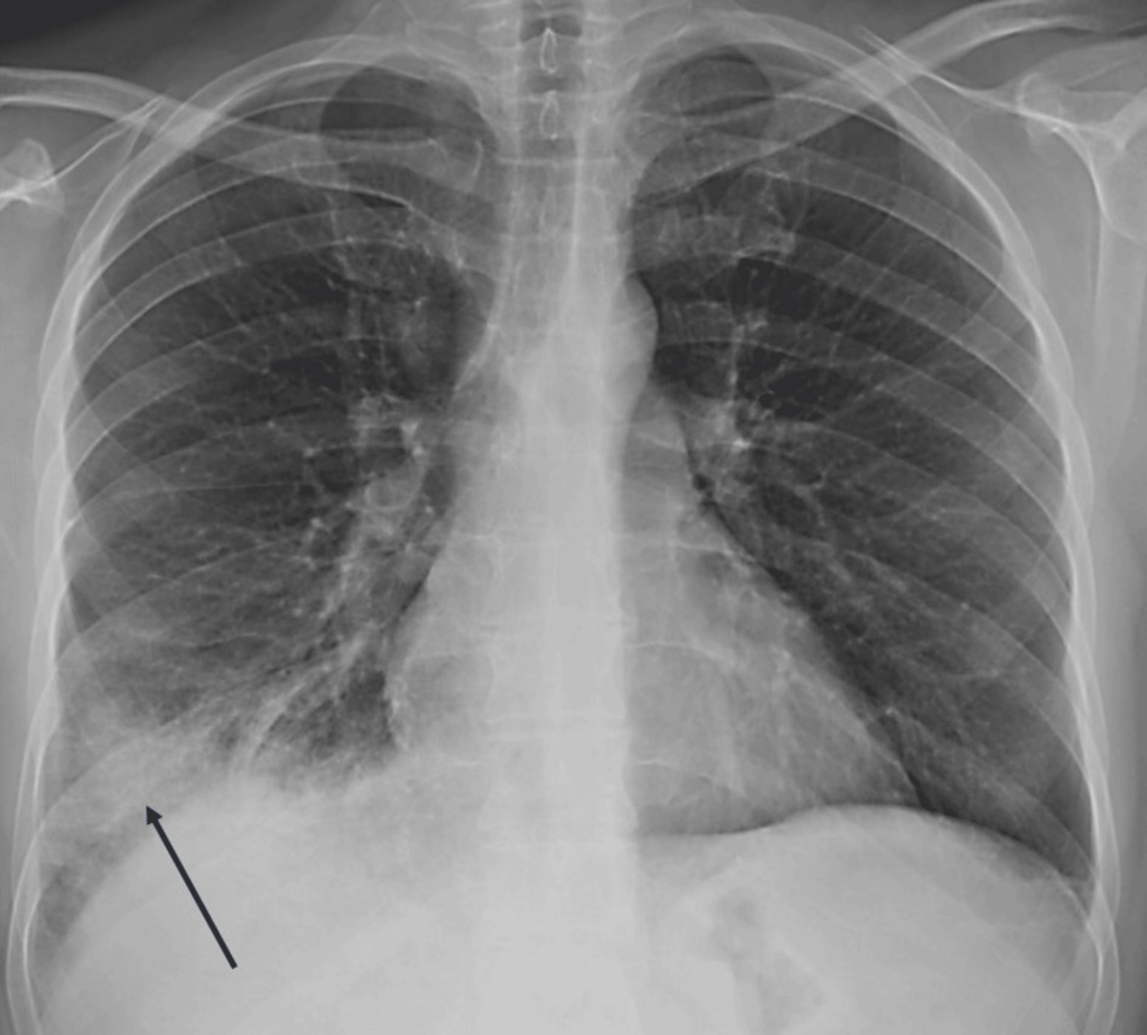
Chest X-ray on admission showing confluent alveolar infiltrates in the right lower lung field with air bronchograms (arrow), consistent with right lower lobe pneumonia.

On initial evaluation, the patient was hemodynamically stable but exhibited low-grade fever and oxygen saturation of 90% in ambient air. Coarse crackles were auscultated in the right middle and lower lung fields. The cardiac examination and the remainder of the physical examination, including assessment of peripheral lymph nodes, were unremarkable. A chest X-ray (Figure 1) revealed confluent alveolar infiltrates in the right lower lung field (arrow) with air bronchograms, finding consistent with right lower lobe pneumonia.

The patient’s laboratory findings revealed mild leukocytosis (11.2 K/μL), significantly elevated C-reactive protein (12.5 mg/dL), and an increased erythrocyte sedimentation rate (50 mm/h), consistent with an acute inflammatory process. These results are summarized in Table 1. His pneumonia severity index score was 38, placing him in Class II, where outpatient treatment would generally be considered appropriate. However, due to the persistence of fever and the presence of pleural effusion, the patient was admitted for further evaluation.

**Table 1:** Laboratory findings of the patient This table summarizes the laboratory results of a patient with right lower lobe pneumonia, including mild leukocytosis, elevated C-reactive protein (CRP), and increased erythrocyte sedimentation rate (ESR), all indicative of an acute inflammatory response.

Parameter	Patient’s Value	Reference Range	Units
Leukocyte count	11.2	4.5–10.5	K/μL
C-reactive protein (CRP)	12.5	<0.5	mg/dL
Erythrocyte sedimentation rate (ESR)	50	<36	mm/hr

**Figure 2:**
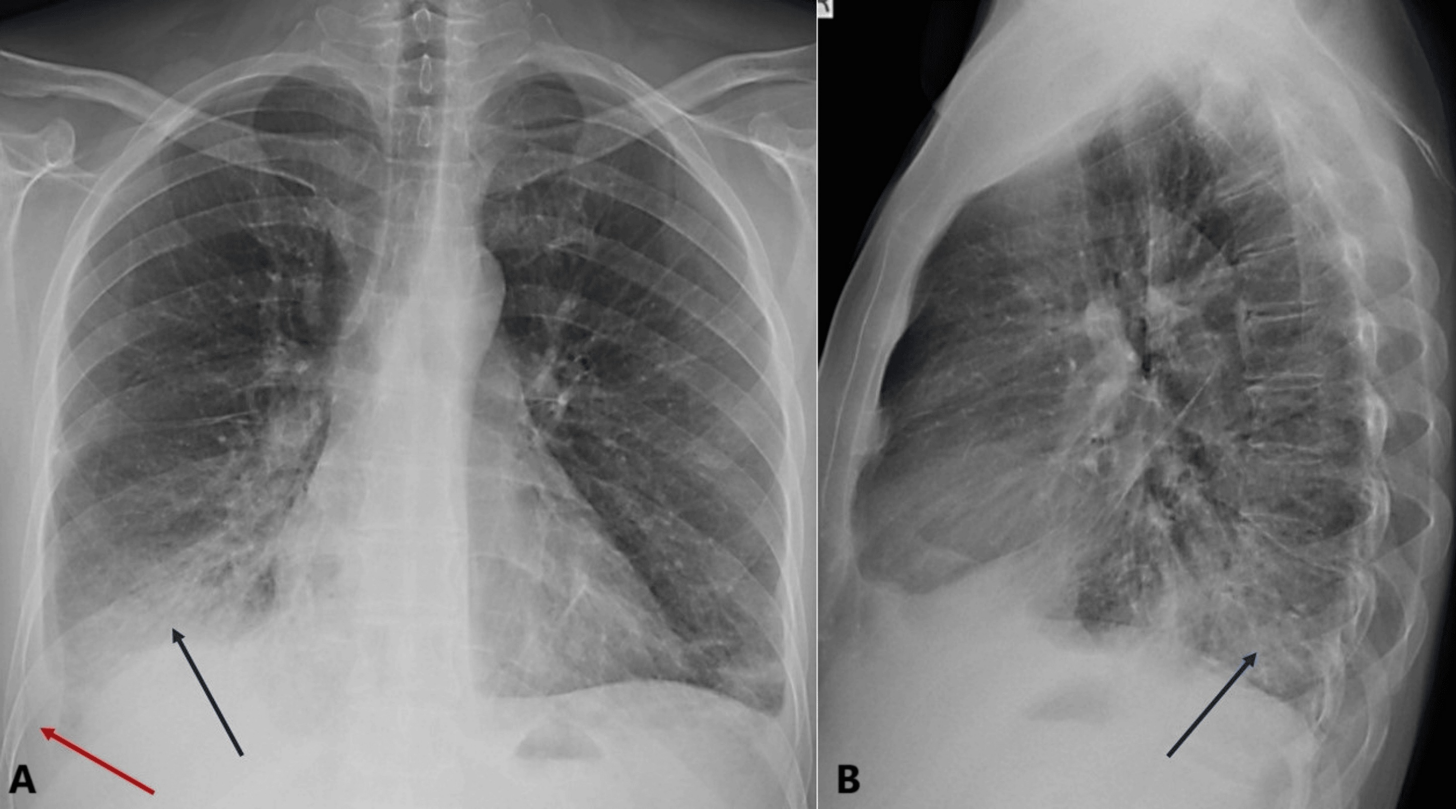
Chest X-ray on day 4 of hospitalization (A) Posterior-anterior view showing worsening right lower lobe infiltrates (blue arrow) and pleural effusion (red arrow), indicating progression of the pneumonia despite initial antibiotic therapy. (B) Lateral view highlighting the same findings (blue arrow), further demonstrating the disease progression.

Upon admission, the patient remained febrile (38.8°C) and required supplemental oxygen via nasal cannula. Antimicrobial therapy with ceftaroline and levofloxacin was initiated for severe community-acquired pneumonia, along with nebulized bronchodilator therapy. By day 4 of hospitalization, the patient’s fever persisted. A repeat chest X-ray (Figure 2) demonstrated worsening infiltrates (blue arrow) and pleural effusion (red arrow).

**Figure 3:**
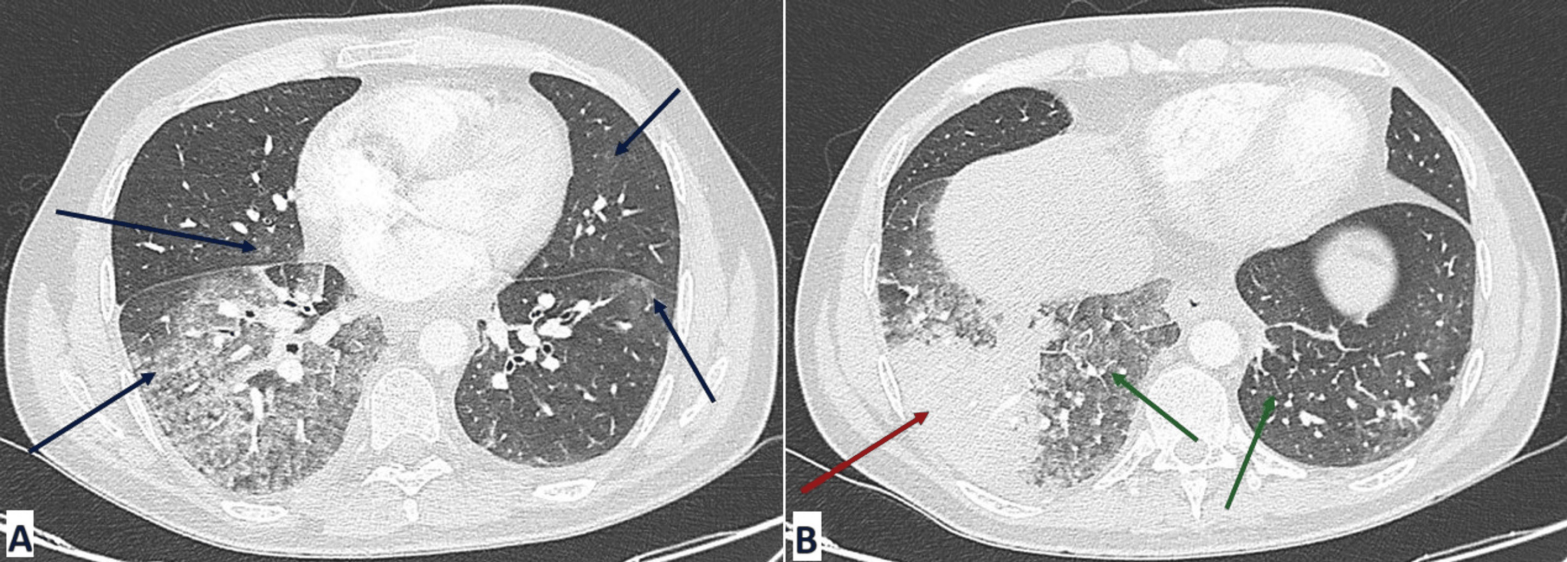
Chest CT scan on day 4 in lung window (A) Axial section at the mid-lung level showing diffuse ground-glass opacities (blue arrows) consistent with severe pneumonia. (B) Axial section at a lower lung level from the same scan, demonstrating consolidation in the right lower lobe (red arrow), and mild interlobular septal thickening (green arrows), further highlighting extensive bilateral lung involvement.

Computed tomography (CT) of the chest (Figure 3) revealed diffuse ground-glass opacities involving at least four lobes (blue arrows), consolidation in the right lower lobe (red arrow), and mild interlobular septal thickening (green arrows).

Diagnostic thoracentesis revealed a lymphocytic (65% lymphocytes) exudative pleural effusion with a lactate dehydrogenase (LDH) to serum LDH ratio of 0.95 and a pleural fluid to serum protein ratio of 0.62. Detailed pleural fluid and serum parameters are presented in Table 2.

**Table 2:** Pleural fluid analysis This table provides the pleural fluid parameters from the diagnostic thoracentesis. The findings suggest a lymphocytic exudative effusion with elevated lactate dehydrogenase (LDH) and protein ratios.

Parameter	Pleural Fluid Value	Reference Range	Units
Lactate dehydrogenase	190	135-225	U/L
Glucose	86	74-103	mg/dL
Total proteins	4.74	6.4-8.3	g/dL
pH	7.42	>7.20	-
Adenosine deaminase	7.9	<20	U/L
Effusion LDH to serum LDH ratio	0.95	-	-
Effusion proteins to serum proteins ratio	0.62	-	-

Given the persistence of fever for nine days despite the administration of broad-spectrum antibiotics, further diagnostic testing was pursued. An autoimmunity panel, serology for hepatitis B, hepatitis C, and HIV, as well as quantitative immunoglobulin measurements, were all normal. A Mantoux test measured 0 mm. Samples were obtained from the upper airway for film array PCR, from pleural effusion for cultures and PCR, and through bronchoscopy with mini-bronchoalveolar lavage (mini-BAL). Cultures and PCR from mini-BAL and pleural effusion were negative for acid-fast bacteria, fungi, and common bacterial pathogens. However, PCR testing detected adenovirus in all three specimens - upper airway, pleural effusion, and mini-BAL- confirming a viral etiology of pneumonia.

**Figure 4:**
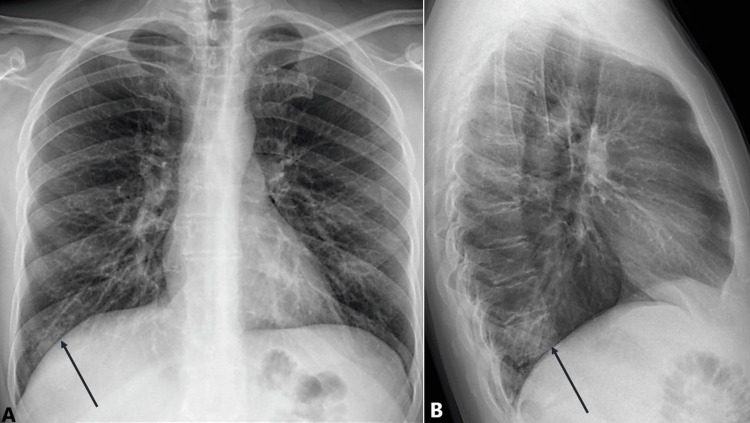
Chest X-ray at 3-week follow-up (A) Posterior-anterior view showing minimal residual findings (black arrow), reflecting near-complete resolution of the pneumonia and pleural effusion. (B) Lateral view confirming no significant abnormalities (black arrow), demonstrating the patient’s recovery.

Antimicrobial therapy was continued to address the potential for secondary bacterial infections, but the primary treatment strategy emphasized careful monitoring and supportive care. By day 6 of hospitalization, the patient showed significant clinical improvement, and he was afebrile by day 7. He was discharged in a stable condition without supplemental oxygen. At a three-week outpatient follow-up, the patient reported no residual symptoms, and his chest X-ray (Figure 4) was near normal, showing minimal residual findings (black arrow).

## Discussion

Adenovirus is a common cause of acute febrile illness and respiratory infections, typically with a favorable prognosis, as the infection is generally self-limiting in immunocompetent individuals. Severe outcomes, however, are more likely in neonates and immunocompromised individuals [12]. While adenoviral pneumonia is well-documented in pediatric populations [13], it is less commonly reported in adults, and when it does occur, it rarely presents with radiologic findings resembling bacterial infections [8], as seen in our patient. The most common radiologic pattern associated with adenoviral pneumonia is diffuse bilateral interstitial shadowing, which is consistent with typical viral pneumonia [14].

In contrast, lobar consolidation, a pattern often associated with bacterial infections, has been observed in approximately one-fourth of patients with adenoviral pneumonia [8]. Our case aligns with this reported pattern of adenoviral pneumonia, but the definitive occurrence is not known, as large-scale studies on viral pneumonia are not always available due to the challenges in detecting these infections. In addition, our case highlights the diagnostic challenge posed by the overlap between adenovirus and bacterial pneumonia, particularly because adenoviral pneumonia can present with a wide range of radiologic findings. These can range from typical viral patterns to more atypical presentations, such as lobar consolidation and pleural effusion, which are often more suggestive of bacterial infection. This underscores the need for a comprehensive diagnostic approach, particularly in severe cases that do not respond to initial antibiotic therapy.

What is particularly novel in our case is the successful isolation of adenovirus in the pleural effusion using PCR, a finding that has only been reported previously in BAL, bronchial secretions or upper-airway specimens [15, 16]. This case is significant because it demonstrates that adenovirus can be present in pleural effusion, which has typically been considered a less likely site for viral isolation. This finding not only confirms the viral etiology but also emphasizes the importance of considering adenovirus in the differential diagnosis of severe pneumonia, especially in cases that present with pleural effusion. As we have shown in our case, comprehensive diagnostic testing, including PCR on pleural fluid, BAL and upper-airway specimens, is essential in establishing the correct diagnosis in challenging cases not responding in empiric therapy [17].

PCR testing has become an indispensable tool in distinguishing between viral and bacterial pneumonia, guiding appropriate treatment, and reducing the need for unnecessary antibiotic escalation [18]. This is crucial, as the increasing identification of respiratory viruses such as respiratory syncytial virus, human metapneumovirus, parainfluenza virus, human rhinovirus, and human coronavirus as causes of community-acquired pneumonia in previously healthy adults further complicates diagnosis. These viruses often present with clinical and radiologic features overlapping with bacterial infections, which makes timely and accurate viral identification critical for appropriate patient management [18].

Moreover, in cases like ours, where the patient does not respond to conventional antibiotic therapy, it is vital to consider viral pathogens such as adenovirus and explore all possible diagnostic avenues, including PCR testing and viral array techniques. This ensures that unnecessary antibiotics are avoided and prompts further investigation into potential undiagnosed immunocompromise in the patient, which could explain the atypical severity of the pneumonia [19].

In conclusion, our case emphasizes the importance of not limiting the differential diagnosis of severe lobar pneumonia to bacterial pathogens such as *Streptococcus pneumoniae*, but also considering viruses like adenovirus, even in the presence of parapneumonic pleural effusion. Early identification of adenovirus allows for more appropriate management, avoiding unnecessary antibiotic escalation, and may lead to further investigations into immunocompromised states that could predispose patients to more severe presentations of viral infections [20].

## Conclusions

This case underscores the importance of broadening the differential diagnosis for severe pneumonia, even when it presents as lobar consolidation and pleural effusion, particularly when conventional antibiotic therapy fails. Adenovirus, although more common in children, should be considered in young adults. Our case highlights the novel detection of adenovirus from pleural effusion using PCR, an approach previously limited to bronchoalveolar lavage or upper airway specimens. While promising, this finding requires validation in larger cohorts. This emphasizes the value of advanced diagnostic methods, such as PCR and viral arrays, in ensuring accurate identification of viral pathogens. Early detection allows for tailored management, prevents unnecessary antimicrobial escalation, and prompts investigation for potential undiagnosed immunocompromised states, ultimately improving patient care.
